# In-situ detoxification of schedule-I chemical warfare agents utilizing Zr(OH)_4_@W-ACF functional material for the development of next generation NBC protective gears

**DOI:** 10.1038/s41598-021-03786-8

**Published:** 2021-12-24

**Authors:** Mohammad Imran, Virendra V. Singh, Prabhat Garg, Avik Mazumder, Lokesh K. Pandey, Pushpendra K. Sharma, Jyotiranjan Acharya, Kumaran Ganesan

**Affiliations:** Defence Research and Development Establishment, DRDO, Jhansi Road, Gwalior, 474002 India

**Keywords:** Environmental sciences, Chemistry, Materials science, Nanoscience and technology

## Abstract

Chemical warfare agents (CWAs) have become a pivotal concern for the global community and spurred a wide spectrum of research for the development of new generation protective materials. Herein, a highly effective self-detoxifying filter consisting of in-situ immobilized Zirconium hydroxide [Zr(OH)_4_] over woven activated carbon fabric [Zr(OH)_4_@W-ACF] is presented for the removal of CWAs. It was prepared to harness the synergistic effect of high surface area of W-ACF, leads to high dispersion of CWAs and high phosphilicity and reactivity of [Zr(OH)_4_]. The synthesized materials were characterized by ATR-FTIR, EDX, SEM, TEM, XPS, TGA, and BET surface area analyzer. The kinetics of  in-situ degradation of CWAs over Zr(OH)_4_@W-ACF were studied and found to be following the first-order reaction kinetics. The rate constant was found to be 0.244 min^−1^ and 2.31 × 10^−2^ min^−1^ for sarin and soman, respectively over Zr(OH)_4_@W-ACF. The potential practical applicability of this work was established by fabricating Zr(OH)_4_@W-ACF as reactive adsorbent layer for protective suit, and found to be meeting the specified criteria in terms of air permeability, tearing strength and nerve agent permeation as per TOP-08-2-501A:2013 and IS-17380:2020. The degradation products of CWAs were analyzed with NMR and GC–MS. The combined properties of dual functional textile with reactive material are expected to open up new exciting avenues in the field of CWAs protective clothing and thus find diverse application in defence and environmental sector.

## Introduction

The new innovative developments in science and technology and sophistication of equipment for delivery make the chemical warfare agents (CWAs) as a most vulnerable threat for military and civilians both from terrorist groups as well as by sovereign states inspite of the presence of a well established Organization for the Prohibition of Chemical Weapons (OPCW) international treaty^1–4^. CWAs are one of the most brutal extremely toxic chemicals created by man and can be deployed in the form of gas, liquid or aerosol or powder forms^5–8^. Nerve agents are regarded as the most dangerous chemical weapon among all CWAs, primarily because of their high toxicity, rapid action following exposure and human sensation inability. Nerve agents has excellent capability to form irreversible phosphonate ester bonds with acetylcholineseterease (AChE) enzyme, which leads to the breakdown of nervous system and results to painful death of the individual^9^.

Recent protection trends have shifted to the development of multifunctional, lightweight, in-situ self-decontaminating active material based protective gear by imparting functionalities to textile materials, which could help to defend first responders and the general public from CWAs scenarios^10–12^. The rapid translation of research activities into real-world defence applications will be aided by new technological innovation that leads to the production of novel materials and a convergence of material and textile science. This potpourri of technologies will be instrumental in addressing the threat posed by CWAs^13^. With the inclusion of dual functional materials, textiles can extend the functionalities with various functions and provide an excellent opportunity to extend the landscape of protective textile with enhanced performance^14–16^. Till date, the most common protective media/fillers currently used for Nuclear, Biological and Chemical (NBC) are carbon-based materials such as activated carbon powder, granular activated carbon, impregnated carbon, and activated carbon spheres. These adsorbent materials are still being used in the development of protective ensemble in particular for CWAs, however, plethora of materials have been explored as an adsorbent to overcome the CWAs detrimental effects and allow the first responder to be effective with least amount of deterioration in operational performance, such as inorganic materials, activated carbon fabric (ACF)^17^, zeolites^2, 18^, porous clay^19^, silica gels^20^, activated carbon^21,22^, and metal organic frameworks (MOF)^23–26^.

Metal organic frameworks (MOFs) have been extensively studied as a potential candidate as an adsorbent for catalytic detoxification of CWAs owing to their high surface area and reactivity when compared to traditional adsorbents, making them a potential candidate for catalytic detoxification of CWAs. However, utilization of the MOFs does not allow for its easy application in the real world owing to its less thermal and moisture stability than the carbon material^24, 27,28^. Expensive precursor material for syntheis of MOFs and lower thermal and hydrolytic stability when compared to carbon based adsorbents severely restricts the industrial realization of MOFs^29^. Another key problem for most of the nanomaterials like porous organic framework (MOFs, COFs etc.), graphene etc. is being in powder form, which make its use in the protective ensemble complicated and hinders its scale up. This drawback can be addressed by using pelletization technique^30, 31^, however, this process leads to increased overall costs compared to commonly used adsorptive materials. Among the different adsorbent materials, activated carbon-based material has been extensively used as versatile adsorbent material for the removal of toxic gases and vapors^32,33^. Because of its specific adsorbing capabilities, high surface area, easy availability, long lifespan, and low cost, it is commonly used as an adsorbent for air-filtration in both military and civilian applications^33–35^. Amidst the above properties, through physical adsorption mechanism on carbon, CWAs are adsorbed on the surface of adsorbent material and can be desorbed readily by either heat or vacuum, which leads to secondary environmental contamination. Moreover, the carbon-based materials have certain drawbacks such as lack of reactivity, high pressure drop, low exposure area, low adsorption capacity etc. Thus, detoxification of CWAs over the protective materials/filters presents a major technological challenge and hence there is a pressing need to develop suitable sorbent materials that can efficiently capture and degrade the CWAs without jeopardising operating textile properties. Activated carbon fibres (ACF), thanks to their excellent absorbent potential, high surface area, unique pore structure and easy accessibility for various types of pores, make it as an attractive prospective adsorbent material for removing CWAs over traditional adsorbents^36–38^. When compared to granular activated carbon, ACF has a higher rate of matter transfer as large number of micropores are open directly on the surfaces^39^. This is especially important in protective equipment, where the reduced weight leads to a lower heat burden and improves the ease of breathing in respirators^33,40,41^. In addition to this, the activated carbon lacks the property of flexibility and after adhesion on protective textile it losses its surface area when compared to ACF^42^. The fabric texture of ACF permits the creation of filters and other protective gears with low-pressure dips, low-velocity flow, and two-dimensional structures, allowing compounds to stay for a long time^43^. ACFs have micropores readily available on the surface of textiles for adsorbate gas, unlike most types of activated carbon, which have a ladder-like pore structure^44^. Since, nerve agents have a P–X bond (X = halogen, nitriles, etc.), the usual method of detoxifying OP-based CWAs is to hydrolyze the P–X bond^8, 45^. Unimpregnated carbon-based adsorbent like GAC and ACF, can adsorb the CWAs however, do not have ability to decompose it^2^, and due to their lack of reactivity, such adsorbents may cause secondary contamination in the environment, even after several days of treatment. Therefore, various reactive catalysts, particularly zirconium hydroxide [Zr(OH)_4_] has attracted the scientific community interest due to their two-dimensional square lattice linked by a double hydroxyl bridge, yielding a stoichiometric Zr(OH)_4_ molecule^46–49^. Since, Zr(OH)_4_ molecule contain both bridging and terminal hydroxyl groups, hence, detoxification capability of hydrolyzing the P–X bond is enhanced^49–52^. The poor processability and handling of reactive material powders prevents their widespread usage in this application. To address this, metal oxide and other nanoparticles have been immobilised on the surface of ACF using several methods including physical spraying^39,53^ direct impregnation^15,54^, in-situ growth on fiber, electrospinning, etc^55,56^. Regardless of our sincere efforts, so far developed protective suits for CWAs has not kept same pace with progress on general methods for CWAs destruction and still it relies on the adsorptive removal, which can cause secondary contamination in the environment. To address the above technological challenge, for the efficient capture and degradation of CWAs without compromising operational textile parameters the present work was undertaken in this study. Based on the above background, to develop the new class of modified protective fabrics aiming to be the alternative for the conventional ACS and charcoal-based filter fabric for NBC suit, utilising the unique properties of ACF with the reactive material Zr(OH)_4_ was explored which can improve the contact between CWAs to the reactive site of Zr(OH)_4_@W-ACF, leading to enhanced degradation of CWAs sarin and soman. Moreover, the integration of in-situ generated Zr(OH)_4_ on the surface of ACF was also demonstrated. To the best of our knowledge, the zirconium hydroxide coated fabric for the degradation of live CWAs has not been reported yet. In this paper, we improve the material and surface properties of the ACF in order to fully realise Zr(OH)_4_@W-ACF protective fabric as a CWAs filter fabric. The degradation of CWAs sarin and soman on Zr(OH)_4_@W-ACF was performed at ambient temperature without using any buffer solution as the use of buffer solution is not feasible in field condition in particular application like protective suits^57^. The resulting Zr(OH)_4_@W-ACF combines the attractive properties of W-ACF for adsorption and Zr(OH)_4_ for the degradation of CWAs sarin and soman. Kinetic rate constants (K) and half life (*t*_1/2_) values for CWAs sarin and soman were determined. The hydrolysis products were also identified by Gas chromatography-mass spectrometer (GC–MS) and Nuclear Magnetic Resonance (NMR) analyses. Surface characterization of Zr(OH)_4_@W-ACF was performed using Fourier Transform Infra Red-Attenuated Total Reflection (FTIR-ATR), Scanning Electron Microscopy (SEM), X-ray Diffraction (XRD), X-ray Photoelectron Spectroscopy (XPS), Transmission Electron Microscopy (TEM), Raman, and Thermogravimetric Analysis (TGA). By utilizing this Zr(OH)_4_@W-ACF the protective suits will be light in weight comfortable and the surface area of Zr(OH)_4_@W-ACF will not be compromised as it can be directly used as filter fabric which is not possible in the case of activated carbon sphere (ACS) and charcoal powder (needs to be deposited on fabric using adhesive). Furthermore, practical applicability of this work was also established by fabricating as reactive adsorbent layer to make a protective suit and some textile parameters like tensile strength, air permeability and nerve agents tests are also carried out to confirm its suitability for the fabrication of protective suits.

## Experimental section

### Materials

All the reagents including Tributylphosphate (TBP), acetonitrile (ACN), ethyl acetate, hexane, toluene, zirconyl chloride octahydrate (ZrOCl_2_. 8H_2_O), NaOH were purchased from Sigma Aldrich, India and were used without further purification. Woven activated carbon fabrics (W-ACF) were sourced from the commercial market. Chemical warfare agents (CWAs) sarin and soman were taken from schedule-I OPCW declared facility of our establishment and the quality of these agents was confirmed by ^1^H NMR (i.e. purity of sarin and soman > 99%). CWAs are extremely lethal compounds that need to be handled in a fume hood using NBC based personal protective devices and CWA decontamination system with appropriate protective measures.

### Material characterization

GC–MS analyses were carried out using an Agilent 7890 N gas chromatograph with a 5975 N mass-selective detector in the electron ionisation (EI) mode (Agilent Technologies, USA). In gas chromatography analyses, an HP-5 (Agilent technologies) capillary column (30 m × 0.25 mm I.D. × 0.25 m film thickness) was employed for separation. Splitless mode was used to analyse the samples, with a splitless time of 2 min and an injector temperature of 250 °C. During complete analyses, the temperature of the EI source was held at 230 °C, the ionisation energy was 70 eV, and the quadrupole temperature was 150 °C. During the analysis, GC oven was programmed from 50 to 280 ºC alongwith ramp-1 with an increment of 20 ºC/min upto 230 ºC and ramp-2 with 30 ºC/min upto 280 ºC with a hold-time 5 min at 280 ºC. The samples were injected into the injector port of the GC system using an auto-injector (Model: Agilent 7693A) and the injection volume was fixed to 1 μL. The selective ion monitoring (SIM) technique was used to conduct CWAs quantification studies. In SIM mode analysis, the dwell duration was 100 ms, and the scan range was 35 to 550 m/z (3.47 scans per second). MassHunter software of GC–MS was utilized for the characterization of GC–MS spectra.

Nitrogen (N_2_) adsorption–desorption isotherms were measured at − 196 °C with an ASAP 2020 (Micromeritics). The samples were degassed to a continuous vacuum (10^–4^ Torr) at 90 °C before analysis. The adsorption isotherms were used to calculate the Braunauer, Emmet, Teller (BET) surface area.

X-Ray-diffractograms of the materials were measured on a Rigaku 5th Generation X-Ray Diffractometer (Model No. Mini Flex 600) paired with high speed D/teX detector, using CuK_α_ radiation (λ = 0.154 nm) over the range of 10–60°.

Structural characterization and elemental composition of the W-ACF and Zr(OH)_4_@W-ACF were carried out using Scanning electron microscope of ICON ANALYTICAL (Manufacture: FEI; Model Quanta 200) with electron dispersive X-ray spectroscopy (EDX) at an acceleration voltage of 5 keV.

ATR-FTIR spectra of the W-ACF and Zr(OH)_4_@W-ACF were condcuted with Bruker Alpha II Comapct FT-IR spectrometer, USA.

**WARNING:** CWAs are extremely hazardous and lethal compounds. They should only be used in designated laboratories by personnel trained in safe-handling and with immediate access to medical support. Individual protective equipment (NBC suit, gloves, overboots, NBC respiratory mask, NBC Canister) must be worn for protection against CWAs. It is responsibility of all users to follow the good laboratory practices while handling the CWAs in the laboratory.

### In-situ surface modification of woven activated carbon fabric with Zr(OH)_4_

Prior to in-situ surface modification of woven ACF (W-ACF), pre-activation was carried out at 110 °C for 5 h to remove any moisture entrapped inside the pore of W-ACF. After activation, the W-ACF was kept in desiccators to avoid any exposure to moisture. The different weight percentage Zr(OH)_4_ (ranging from 2 to 12%,w/w) was incorporated over the W-ACF by dissolving appropriate amount of ZrOCl_2_.8H_2_O in water. Several methods are available for the surface modification of the W-ACF including microwave^24^, sol–gel method^58^, impregnation^53,59^, ultrasonication^24,60^ etc. Among these, the impregnation method was preferred for W-ACF surface modification because of the woven nature of the fabric which has convenient pore size distribution to entrapped the Zr(OH)_4_ over the surface of W-ACF^53,59^. After incorporation of ZrOCl_2_.8H_2_O on the surface of W-ACF, the samples were washed with a copious amount of water to remove any unbound chemical species followed by its drying at 110 °C for 5 h. Furthermore, The ZrOCl_2_.8H_2_O coated W-ACF was passed through the aqueous solution of respective strength of NaOH, resulting in the in-situ production of Zr(OH)_4_ on the ACF surface. The prepared woven Activated Carbon Fabric with Zr(OH)_4_ [Zr(OH)_4_@W-ACF] samples were washed with Milli-Q water three times to eliminate any unreacted NaOH. Subsequently, the prepared Zr(OH)_4_@W-ACF samples were dried at 100 °C for 6 h and stored in desiccators to avoid any moisture exposure before being used in the degradation study of CWAs Sarin and Soman.

### Selection of solvent for extraction

In order to ensure the efficient extraction, organic solvent hexane, toluene, ACN and ethyl acetate were evalauted for extraction of CWAs sarin and soman. For this extraction efficiency test, 250 μl of 400 ppm sarin and soman were spiked over W-ACF system and after one minute interval it was extracted with different organic solvents by varying extraction time from 5 to 20 min.

### Kinetic experiments with Zr(OH)_4_@W-ACF and W-ACF

To study the degradation kinetics of the nerve agents sarin and soman, a test strip of Zr(OH)_4_@W-ACF and its corresponding control sample W-ACF were cut into a strip having weight 0.15 g (fixed for specimen and control) with their approximate dimension 1.5 × 3.6 cm^2^ for Zr(OH)_4_@W-ACF and 1.5 × 4.5 cm^2^ for W-ACF. The difference in dimensions can be attributed to the change in weight after impregnation, as the weight of the fabric is drastically increased. Standard stock solutions of 1.0 mg/mL (1000 ppm) of the CWAs sarin and soman, and tributylphosphate (TBP), were prepared in ACN.

### Standard spiking solutions

As discussed above, standard stock solutions of 1.0 mg/mL (1000 ppm) of the CWAs sarin and soman, and tributylphosphate (TBP) were prepared in ACN and diluting it as needed. A working standard of 0.4 mg/mL (400 ppm) of sarin and soman was prepared by diluting the corresponding stock solutions of each analyte in ACN and the chromatographic standard with ethyl acetate to obtain desired concentration and preparing the corresponding control samples. 250 μl of 400 ppm sarin and soman were spiked over 0.15 g of Zr(OH)_4_@W-ACF and control W-ACF system in a stopper glass tube. Multiple samples along with control were kept at room temperature (25 ± 1 °C). After a fixed time intervals the contaminated samples were extracted with ethyl acetate and the extracts were analyzed for residual amounts of CWAs using a GC–MS.

### Protocol for kinetic degradation studies of CWAs Sarin and Soman over Zr(OH)_4_@W-ACF

Sarin having volume 250 µl of 0.4 mg/mL was directly spiked over the surface of each testing specimen and their corresponding control sample simultaneously over time intervals 02, 04, 06, 08, 10, 12, 14, 16, 18 and 20 min respectively. For soman, 250 µl of 0.4 mg/ml was directly spiked over the surface of each test specimen and their corresponding control sample simultaneously over time intervals 20, 40, 60, 80, 100, 120 min, respectively. To identify the degraded product, ACN was used to extract the degradation product, which was then sampled and subjected to GC–MS analysis (after silylating the extract with Bis(trimethylsilyl)-trifluoro acetamide (BSTFA).

### Test conditions for NMR analyses

The solvent was optimised by performing NMR measurements in the appropriate deuterated solvent under field frequency locked conditions (acetone-d_6_, methanol-d_4_, chloroform-d_1_, acetonitrile-d_3_, benzene-d_6_, pyridine-d_5_, dimethyl sulfoxide-d_6,_ and deuterium oxide). A homogenous magnetic field was estabished by manually shimming the sample before each experiment and then utilising the TopShim gradient shimming function in the Topspin 3.2 programme. The Autoshim module of the Topspin programme was utilised to provide adequate stability and magnetic field homogeneity throughout the experiment.

## Results and discussion

### Characterization

**Figure 1:**
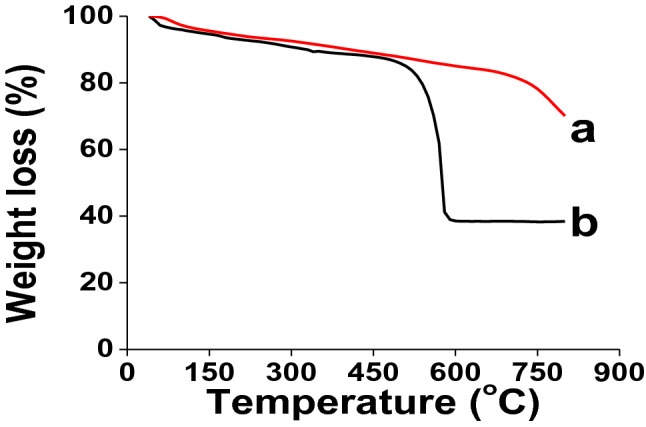
TGA curves of (**a**) W-ACF and (**b**) Zr(OH)_4_@W-ACF.

To characterize the prepared sample of Zr(OH)_4_@W-ACF, different analytical instruments were explored. Thermal gravimetric analyses (TGA) of ACF and Zr(OH)_4_@W-ACF were performed to see the thermal stability of Zr(OH)_4_@W-ACF and its control samples, were measured from 40 to 800 °C with a 10 °C min^-1^ ramp under N_2_ atmosphere (Fig. 1). In the case of ACF, it can be seen that, between 100 and 280 °C, there was a loss of 6.7% of the initial mass with the elimination of moisture from the sample^61,62^. Due to slow thermal decomposition of the organic carbon structure, the second stage of TGA shows around 8% weight loss in the temperature range of 280 to 600 °C^61^. The residual weight at 800 °C for ACF is 70%, and this reflects the higher thermal stability of ACF. The another control i.e. ZrOCl_2_.8H_2_O, TGA study is carried out and presented as Fig. S1. As can be seen from the Fig. S1, the initial weight loss of 23% up to 100 °C is ascribed to loss of water molecules^61,62^. From 100 to 170 °C range, a dehydration step can be attributed to the displacement of hydrochloric acid from the lattice as well as continued removal of co-ordinatedly bound water molecules which is in agreement with previous finding^63,64^. While in the case of Zr(OH)_4_@W-ACF, the initial mass loss of 6% is attributed to bulk water and bound surface or coordinated water species in between 75 and 150 °C. Between 520 and ∼590 ºC, a significant weight loss of 47% is observed and is attributed to bridging and terminal hydroxyl groups forming oxide bonds during crystallization to zirconium dioxide as reported elsewhere^40,65,66^. After 590 °C, there is no further weight loss was observed^40^. These findings are consistent with the values obtained from weight changes. From the above results, it can be concluded that control samples ACF and ZrOCl_2_.8H_2_O, TGA profiles are different than the synthesized Zr(OH)_4_@W-ACF, which confirmed that Zr(OH)_4_ was present over W-ACF.

**Figure 2:**
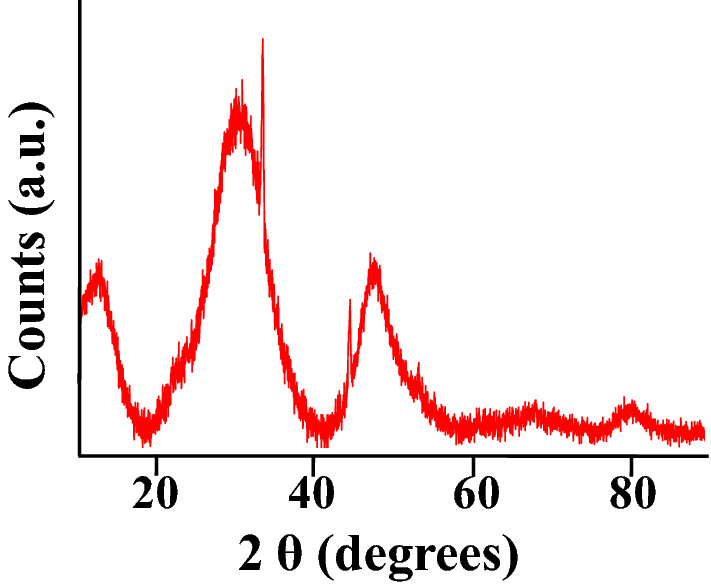
XRD pattern of Zr(OH)_4_@W-ACF.

X-ray powder diffraction (XRD) studies (Fig. 2) were conducted to study the ACF and the in-situ synthesized Zr(OH)_4_@W-ACF crystalline phase. The peaks for the (002) and (100) planes of graphitic carbon are indexed to the XRD diffraction pattern of ACF^34^. The semi-crystalline structure of W-ACF (Figure S2) has been proven by the existence of a large peak around 26.2°, which depicts the superior alignment of disordered graphitic carbon layers to create the crystalline turbostratic structure^34^. The XRD pattern of Zr(OH)_4_@W-ACF showed three broad peaks at 30.1, 50.1, and 64.6° 2θ that clearly indicate that there is formation of amorphous Zr(OH)_4_, as reported elsewhere^50, 67,68^. Being amorphous nature of both the materials, the similar XRD patterns was observed for W-ACF and Zr(OH)_4_@W-ACF with small shifting in 2θ in case of Zr(OH)_4_@W-ACF^69^. The amorphous crystal structure of Zr(OH)_4_ with short-range crystallographic ordering is shown by the broadness of these peaks. These XRD patterns indicated and confirmed the presence of Zr(OH)_4_ over the surface of W-ACF.

**Figure 3:**
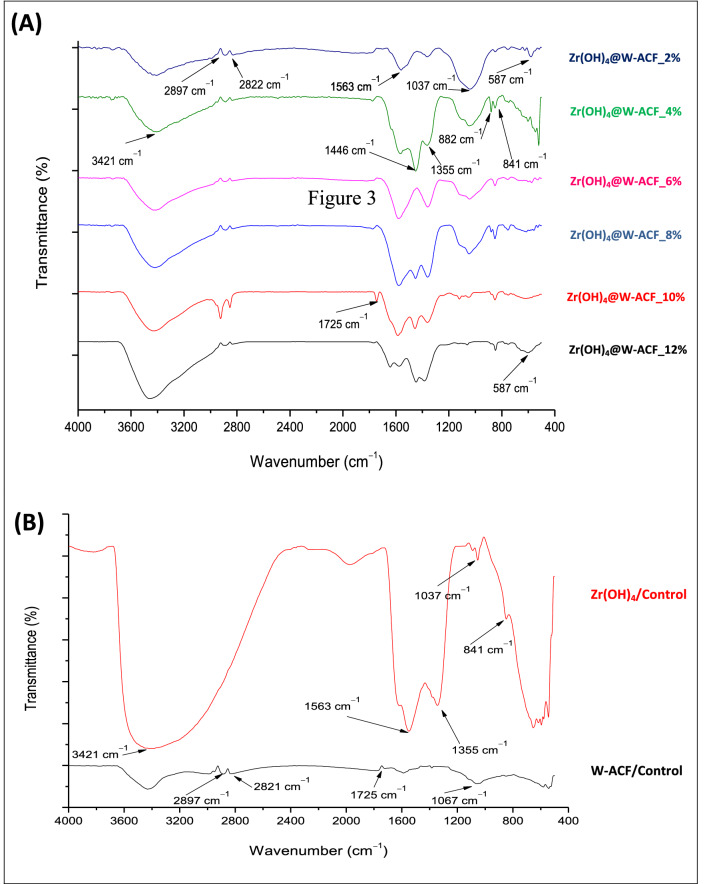
ATR-FTIR spectra of (**A**) different loading percentage of Zr(OH)_4_@W-ACF, and (**B**) Zr (OH)_4_ and ACF.

ATR-FTIR study was also carried out to know the in-situ deposition of Zr(OH)_4_ in the three-dimensional network of W-ACF surface as described in Fig. 3. In case of Fig. 3B, Zr(OH)_4_, the peak at 3437 cm^-1^ is very borad and assigned for hydrogen bonded stretching of Zr-OH, a charecteristic peak for hydrated compound^70^. The absorbance intensity of the hydrogen bonded hydroxyl bond (–OH) is getting increased [Zr(OH)_4_@W-ACF_2% to Zr(OH)_4_@W-ACF_12%] after incresing the impregnation amount, indicating the presence of a rich hydroxyl group on the ACF surface that will be effective towards decontamination of CWA sarin and soman (Fig. 3A). However, in the case of ACF (Fig. 3B) the peak at 3400 cm^−1^ is small and this corresponds to stretching and bending modes of H bonded –COOH group^40^. The peaks at 2897 cm^-1^ and 2822 cm^-1^ are assigned for C–H stretching vibration of –CH_2_ and aromatic C–H stretching vibration, respectively^71, 72^ which is present in both ACF and Zr(OH)_4_@W-ACF while it is absent in Zr(OH)_4_ sample which confirmed the modification of ACF surface. The peak observed in the region of 1563 cm^-1^ for all the samples of Zr(OH)_4_@W-ACF (Fig 3A) are due to the adsorbed moisture and this finding is in agreement with the previous literature^73^.

The IR spectra of W-ACF (Fig. 3B) and Zr(OH)_4_@W-ACF (Fig. 3A) showed small peaks 1725 cm^-1^ that are attributed to –C=O group of ester, caroxylic acid of ACF and this peak is absent in Zr (OH)_4_. The peak at 1725 cm^-1^ is common in W-ACF as well as Zr(OH)_4_@W-ACF, however, due to high intensity of peak at 3400 cm^−1^ this peak got reduced. Moreover, the bands observed at 1446 cm^−1^ and 1355 cm^-1^ are assigned to the complexes of formates and carbonate species which readily form when CO_2_ reacts with free hydroxyl group of Zr(OH)_4_^52^. In Zr(OH)_4_@W-ACF sample, the peak observed at 1037 cm^−1^ is assigned to bending vibration of hydroxyl group of the Zr –OH typically for zirconium hydroxide which is absent in the ACF control sample^74^. This finding also confirms the presence of Zr(OH)_4_ over the W-ACF surface. Due to high intensity of peak at 3421 cm^-1^, the intensity of peak at 1037 cm^-1^ is reduced in the sample Zr(OH)_4_@W-ACF_8% to 12%. The peak at 882 cm^-1^ and 841 cm^-1^ 587 cm^-1^ are attributed to the metal–oxygen stretching modes of Zr-O^70,74^. This peak is also absent in W-ACF, and this could be regarded as evidence for the modification of Zr(OH)_4_ over W-ACF.

X-ray photoelectron spectroscopic (XPS) studies were conducted for W-ACF and Zr(OH)_4_@W-ACF (Figure S3). As can be seen from the XPS survey spectra of W-ACF (Fig. S3A) and Zr(OH)_4_@W-ACF (Fig. S3B), the Zr (3d) peak is absent in W-ACF. The Zr 3d of Zr(OH)_4_@W-ACF spectrum (Fig. S3C) showed two components 3d_5/2_ and 3d_3/2_ which is centered at 182.64 and 184.94 eV, respectively with the appropriate peak area ratio of 3:2^47,75, 76^. Fig. S3E, shows three deconvoluted peaks for the O 1s spectra at 534.48, 531.98 and 529.85 eV corresponding to H–O–H, terminal and bridging OH groups, respectively. The ratio of terminal to bridging OH group shows the ratio of 1: 3.5 which is in agreement with earlier reports and confirms the formation of Zr(OH)_4_^75,77^. The C 1s band of Zr(OH)_4_@W-ACF (Figure S3D) was deconvoluted into three peaks located at 284.98, 286.77 and 289.08 eV, corresponding respectively to the C=C/C–C, C–O and –O–C=O groups^78^.

Figure S4 shows the Raman spectra of W-ACF and Zr(OH)_4_@W-ACF. Figure S4A shows the Raman spectrum of ACF which exhibit two bands at 1330.74 cm^-1^ and 1589.77 cm^-1^ and these are ascribed as D (disorder and defects) and G (sp^2^ graphitic structure) band. After in-situ modification of ACF with Zr(OH)_4_ the peak at 1330.74 cm^-1^ and 1589.77 cm^-1^ are shifted to 1336.20 cm^-1^ and 1594.1 cm^-1^, respectively which confirms the modification of ACF surface^76,79^. Morover, the peak at 147.19 cm^-1^ is assigned to teragonal zirconia while peaks at 518.19 cm^-1^ and 1061.60 cm^-1^ are due to monoclinic form of zirconia in Zr(OH)_4_@W-ACF^70^. The shifting of D and G bands in Zr(OH)_4_@W-ACF samples and appearance of peaks for tetragonal and monoclinic zirconia phase further confirms the in-situ modification of W-ACF surface with Zr(OH)_4._

**Figure 4:**
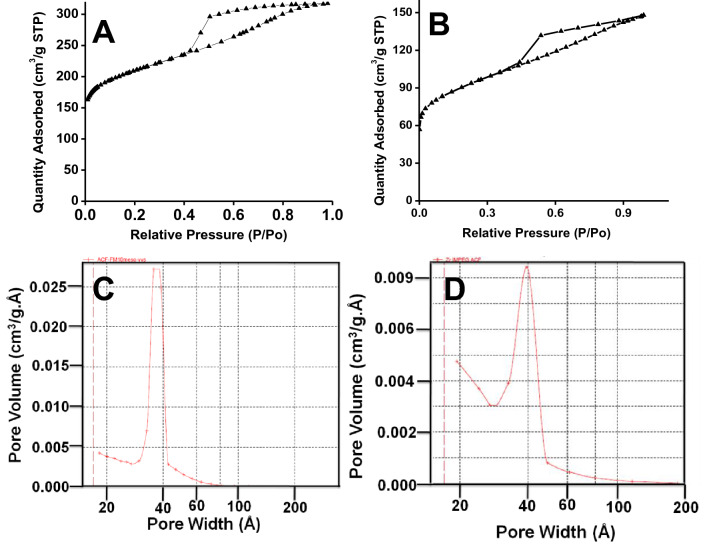
Nitrogen adsorption/desorption isotherm of (**A**) W-ACF and (**B**) Zr(OH)_4_@W-ACF and Pore size distribution of (**C**) W-ACF and (**D**) Zr(OH)_4_@W-ACF.

Nitrogen adsorption–desorption isotherms of Zr(OH)_4_@W-ACF and corresponding control samples were performed to confirm the in-situ immobilization of Zr(OH)_4_ on W-ACF utilising incipient wetness technique that helps to incorporate metal salts within the pore structure of W-ACF. Figure 4 shows the Nitrogen adsorption–desorption isotherms of W-ACF (Fig. 4A) and Zr(OH)_4_@W-ACF (Fig. 4B) exhibited type-IV adsorption hysteresis reflects the presence of mesoporosity with 600 m^2^/g surface area, after impregnation, the surface area of Zr(OH)_4_@W-ACF (Fig. 4B) is reduced to 350 m^2^/g. Impregnation was found to affect the micropore, mesopore structures and surface area of W-ACF samples when surface and pore volume values were emulated. (Table 1).

**Table 1:** Surface area and pore volume data of W-ACF and Zr(OH)_4_@W-ACF.

Sample name	Surface area (m ^2^/g)	Total pore volume (mL/g)	Micropore volume (mL/g)
W-ACF	600	0.491	0.177
Zr(OH)_4_@W-ACF	350	0.228	0.143

Figure 4 C&D shows the pore size distribution of the W-ACF and Zr(OH)_4_@W-ACF, respectively. The mesoporous structure underwent drastic changes from 0.491 mL/g (Fig. 4C) to 0.228 mL/g (Fig. 4D), however, micropore volume is reduced from 0.177 mL/g to 0.143 mL/g (Fig. 4D). This observation can be attributed that impregnate restricting the entrance to micropores. Though active metal component partially restrict the pores, optimization of the impregnates loading is crucial for making the easy access of CWAs molecules toward reactive sites within the mesopores.

**Figure 5:**
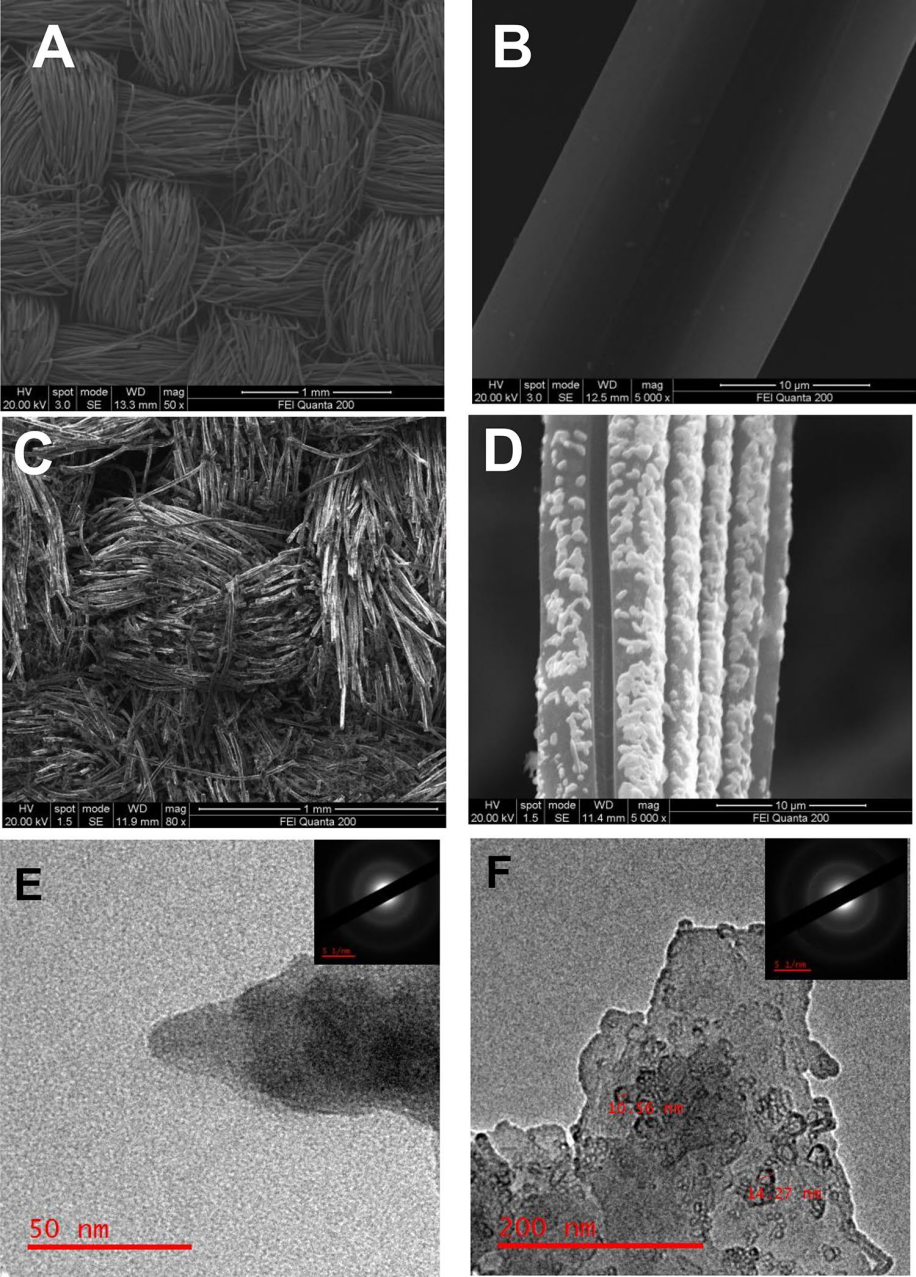
SEM images illustrating the surface of the W-ACF and Zr(OH)_4_@W-ACF: (**A**) W-ACF (low magnification), (**B**) W-ACF (higher magnification), (**C**) Zr(OH)_4_@W-ACF (low magnification) (**D**) Zr(OH)_4_@W-ACF (higher magnification) (**E**) TEM image of W-ACF and (**F**) TEM image of Zr(OH)_4_@W-ACF.

The surface morphology of the W-ACF and modified W-ACF surface, i.e. Zr(OH)_4_@W-ACF, was studied using scanning electron microscopy (SEM). Figure 5(A, B) shows the SEM image for ACF, showing microstructures of compact bundles of woven threads of fibers, which are entangled with each other, and showing pseudo-two-dimensional network and smooth and even morphology (Fig. 5B)^80^. As can be seen from the SEM image (Fig. 5A), the fasciculus of ACF is having a diameter of around 0.65 mm comprising of a bundle of fibres with an average diameter of around 17 µm. To confirm and compare the presence of Zr(OH)_4_ particles, SEM images of Zr(OH)_4_@W-ACF were also studied. After impregnation of Zr(OH)_4_ over W-ACF and subsequent heat treatment, the surface of W-ACF is not smooth and it shows the presence of a large number of granules on the surface of W-ACF Fig. 5(C, D). In higher magnification (Fig. 5D), it can be seen that the interconnected nodules make porous structure^81^ and such type of structure could significantly increase the effective surface area and improve the chemical conversion^82^. These nodules like microstructure are due to the formation of in-situ Zr(OH)_4_ only as ZrOCl_2_.8H_2_O was added when modifying the W-ACF. TEM analysis was also carried out in order to get more insight about the structures. The chaotic hierarchical porous structure, which incorporates mesopores and micropores, can be seen in Fig. 5E. The great number of white spots between the disordered carbon layers indicates that the ACF has plenty of micropores and mesopores for impregnation. The selected area electron diffraction (SAED) pattern of the W-ACF reveals diffused diffraction rings, indicating that the W-ACF is amorphous in nature. After impregnation with Zr(OH)_4_, it has been observed that small nanocrystal structure (Fig. 5F) having size aprox 10–14 nm and SAED pattern shows the semicrystalline nature of Zr(OH)_4_@W-ACF, which further confirms the modification on W-ACF surface. The particle size distribution histogram (Figure S5) shows the means size of particle is 8.0 nm.

**Figure 6:**
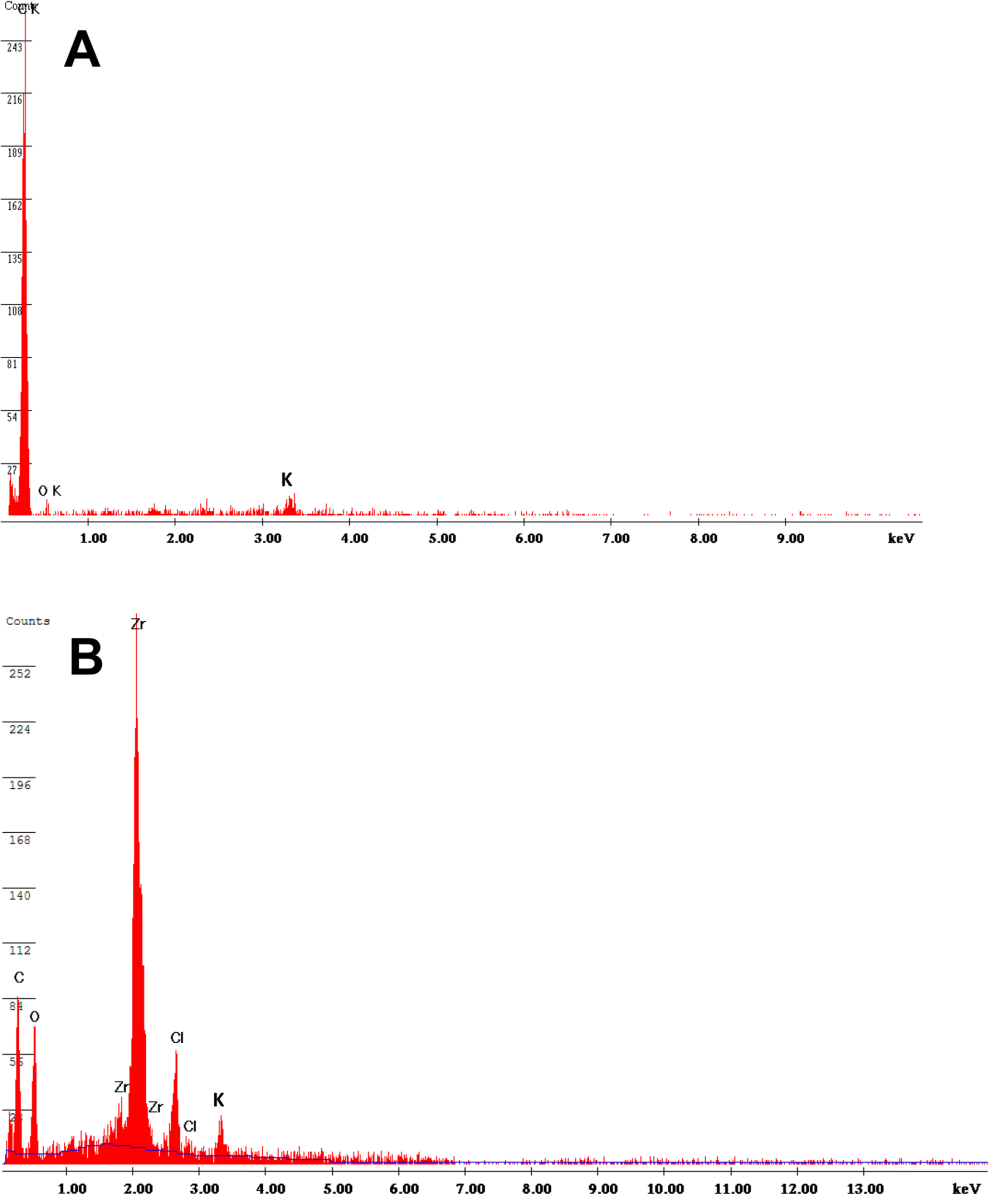
EDX analysis of (**A**) W-ACF and (**B**) Zr(OH)_4_@W-ACF.

Energy dispersive X-ray spectroscopic (EDX) elemental analyses were carried out to determine the chemical composition of W-ACF (Fig. 6A) and Zr(OH)_4_@W-ACF (Fig. 6B) so as to confirm the modification of W-ACF with in-situ grown Zr(OH)_4_ over the surface. In case of W-ACF (Fig. 6A), carbon and oxygen with an atomic percentage of 88.03 and 8.71%, respectively are the main peaks, there was no Zr detected on the W-ACF while in case of Zr(OH)_4_@W-ACF, it shows the presence of zirconium, carbon and oxygen with an atomic percentage of 31.29, 47.63 and 16.88%, respectively which clearly indicate the presence of Zr(OH)_4_ on the surface of W-ACF. It was noticed from the results that the oxygen content increased double in case of Zr(OH)_4_@W-ACF than W- ACF, which will be beneficial to improve the degradation of CWAs. The peak at about 3.3 eV is due to K and this peak is common in both W-ACF and Zr(OH)_4_@W-ACF, which may used during the stabilization process of precursor with KOH. Furthermore, small traces of Cl (4.2%) is due to the use of ZrOCl_2_.8H_2_O for the in-situ syntheis of Zr(OH)_4_ over W-ACF surface.

### Parameters optimization for CWAs degradation on Zr(OH)_4_@W-ACF surface

**Figure 7:**
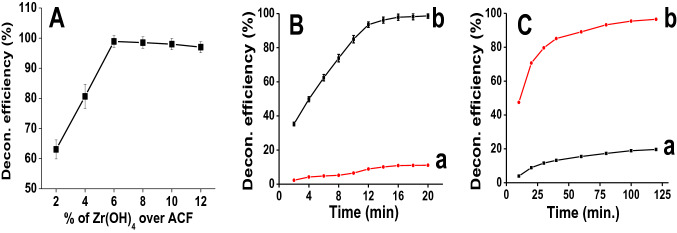
Effect of relevant parameters upon the degradation efficiency of CWA on Zr(OH)_4_@W-ACF: (**A**) Effect of the percentage loading of Zr(OH)_4_, (**B**) Degradation efficiency for CWA sarin- (a) with W-ACF (b) with Zr(OH)_4_@W-ACF, and (**C**) Degradation efficiency for CWA soman- (a) with W-ACF (b) with Zr(OH)_4_@W-ACF.

The key factors controlling the degradation activity of Zr(OH)_4_@W-ACF have been elucidated towards optimizing the degradation of CWA sarin on the modified surface. In order to establish the optimum loading for the degradation, the influence of the various amount of Zr(OH)_4_ loading on W-ACF surface was also studied (Fig. 7A). In general, different loading of Zr(OH)_4_ cause different catalytic activity. Therefore, different loading of Zr(OH)_4_ was carried out on the surface of W-ACF ranging from 2 to 12% (Fig. 7A) and the degradation of sarin was monitored up to 20 min (Fig. 7A). With increase in the Zr(OH)_4_ loadings from 2 to 6 wt%, the percentage degradation of sarin was increased from 63 to 99%. This tendency is attributed to the increase of active metal species which enhances the degradation of CWAs. Subsequently, loading of Zr(OH)_4_ above 8% does not have a significant effect on the degradation of sarin as higher loading percentage of Zr(OH)_4_ may block the pores of W-ACF, which lead to low accessibility of CWA sarin with Zr(OH)_4_^39,83^. Hence, 6% Zr(OH)_4_ loading on W-ACF was employed in all further degradation studies of sarin and soman.

For extraction efficiency test, 250 μl of 400 ppm sarin was spiked on the surface of W-ACF and after one minute contact time, it was extracted with different solvents by keeping the extraction time of 10 min. Results of extraction efficiency with different solvents are summarized in Fig S6. From Fig S6 it has been observed that, the extraction efficiency of ethyl acetate is 94 ± 1.75% when compared to ACN of 89 ± 2%. Hence, ethylacetate was used as extraction solvent for further studies.

After optimizing the solvent, the extraction time for sarin was also optimized with ethyl acetate. Extraction time with ethyl acetate was varied from 5 to 20 min. From the Fig. S7, it is evident that 15–20 min extraction time is sufficient to achieve maximum extraction. Hence, 15 min extraction time was chosen for the entire degradation studies.

**Figure 8:**
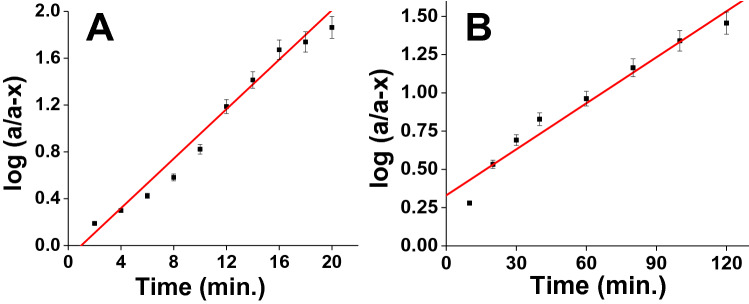
Kinetics of degradation of CWA (**A**) sarin and (**B**) soman on Zr(OH)_4_@W-ACF at ambient temperature.

After optimizing the loading percentage of Zr(OH)_4_ on W-ACF, influence of treatment time upon the efficiency of degradation of sarin (Fig. 7B) and soman (Fig. 7C) were optimized by varying the time from 2 to 20 min with a step of 2 min and 10–120 min with a step of 10 min, respectively. The concentration of degraded sarin and soman over Zr(OH)_4_@ W-ACF were determined at different time interval utilizing GC–MS in SIM mode. The sarin degradation efficiency of the Zr(OH)_4_@W-ACF was enhanced from 35 to 98.6% over this time scale (Fig. 8Bb). In contrast, the W-ACF shows lower degradation efficiency up to 11% in 20 min. Another control sample of NaOH@W-ACF was prepared by impregnating 6% NaOH in W-ACF. After impregnation of NaOH, the prepared sample NaOH@W-ACF was washed with Milli Q water three times to eliminate any unbound NaOH in order to keep the same reaction conditions as in the case of Zr(OH)_4_@W-ACF. The degradation of sarin was studied up to 20 min with a step of 2 min (Figure S8). With NaOH@W-ACF, only 12% sarin removal was achieved in 20 min. The low adsorptive degradation efficiency of NaOH@W-ACF is potentially due to the nature of NaOH. Being an ionic compound, the solubility of NaOH is more in water which lead to the leaching of NaOH from the carbon surface. When compared to W-ACF (Fig. 7Ba), there is 9-folds enhancement in the adsorptive degradation of sarin on Zr(OH)_4_@W-ACF which showed the synergistic effect of highly adsorptive W-ACF and reactive Zr(OH)_4_ material. As can be seen from the Fig. 7Bb, after 18 min of treatment, the degradation of sarin was almost constant, due to saturation of the reactive sites of Zr(OH)_4_ at high treatment time.

While in case of soman, the degradation study was performed from 10 to 120 min with a step of 10 min. As can be seen from the Fig. 7C, with Zr(OH)_4_@W-ACF (Fig. 7Cb) the degradation of soman increased from 47 to 97% over this time scale, however, with W-ACF (Fig. 7Ca) only 20% removal was observed within 120 min. After 120 min, no further significant increment in the degradation with Zr(OH)_4_@W-ACF is achieved that may be attributed due to saturation of active sites. In both the case of CWAs sarin and soman, a dramatic increase in degradation, corresponding to over 98% removal is observed after following the treatment with Zr(OH)_4_@W-ACF. On the other hand, control samples exhibited only 11% and 20% degradation for CWAs sarin and soman, respectively highlighting the necessity of Zr(OH)_4_@W-ACF for the unique interaction between electronically rich phosphate group (Lewis base) of CWAs and electron deficient zirconium cation (Lewis acid) of the Zr(OH)_4_@W-ACF. Thus, the weak physical interaction between the sarin and soman with W-ACF is not sufficient for the removal of CWAs. These data exemplify that the new reactive Zr(OH)_4_@W-ACF offer significantly shorter remediation time than W-ACF, which was achieved by coupling the advantages gained by the open porous structure of W-ACF and the reactive properties of Zr(OH)_4_. The Lewis acid–base interaction between the electrically rich phosphate group of the nerve agents and the electron deficient zirconium cation of the Zr(OH)_4_ results in coordination with the nerve agent and removal of the fluorine atom from the phosphorous centre, yielding pinacolyl methylphosphonic acid which further hydrolyzed in to methyl phosphonic acid in case of soman^84^. In similar manner, sarin also hydrolyzed to isopropylmethyl phosphonic acid and then finally converted to methyl phosphonic acid.

### Kinetics of degradation of CWAs Sarin and Soman on Zr(OH)_4_@W-ACF

Kinetic studies were performed in order to get insight into the reaction mechanism of CWAs sarin and soman degradation on Zr(OH)_4_@W-ACF and W-ACF, a graph was plotted between log (*a*/*a* − *x*) and time as illustrated in Fig. 8; where ‘*a'*, is the initial concentration and ‘*x*' is the degraded amount. As can be seen from the Fig. 7(B, C) with Zr(OH)_4_@W-ACF, initially the degradation of CWAs sarin and soman is fast and it slowed down after several minutes to achieve a steady state exhibiting first order behaviour. This behaviour could be ascribed by the availability of active sites in terms of hydroxyl group, at the initial stages of the reaction, concentration of the active sites (more number of hydroxyl group) is expected to be large, hence, the rate of reaction is high and the slope of the initial portion (linear) of the curve is also high. As the time progresses, the number of available active sites is reduced and formation of reaction product which are co-adsorbed on the active sites leading to a negligible slope and slow degradation rate as reported previously by some of the research groups^85,86^. Linearity of curves shows the kinetics of degradation of CWAs sarin and soman on Zr(OH)_4_@W-ACF (see Fig. 8A and B) follow the first order kinetics^85,87^. The regression coefficient (R^2^) values for the first order kinetic model with CWAs sarin and soman was 0.973 and 0.981, respectively which indicates the applicability of the first order nature of degradation process on Zr(OH)_4_@W-ACF. Rate constant (K) was calculated using the slope of the straight line and half life (t_1/2_) by 0.693/K and are summarized in Table 2.

**Table 2:** Rate constant and half-life of degradation of CWAs on Zr(OH)_4_@W-ACF.

Sample name	CWA	Rate constant (K) (min^−1^)	Half-life (t_1/2_) (min.)
Zr(OH)_4_@W-ACF	Sarin	0.244	2.840
	Soman	2.31 × 10^−2^	30.0

The K values are the average of three runs with ± 5% reproducibility. Initially, the degradation of sarin and soman was found to be very fast which gradually slowed down to a steady state. With Zr(OH)_4_@W-ACF the degradation of sarin, half life (t_1/2_) and rate constant (K) were found to be 2.84 min and 0.244 min^−1^, respectively. While in case of degradation of soman half life and rate constant were found to be 30 min and 2.31 × 10^–2^ min^−1^, respectively, which showed that kinetics of sarin degradation is faster when compared to soman. It is important to note that the half-life for detoxification of sarin using Zr(OH)_4_@W-ACF is 2.84 min, and for soman using the same materials under similar conditions was 30 min. This increased in t_1/2_ with soman when compared to sarin is potentially due to the increasing size of soman which hinders the accessibility of phosphate group to coordinate with zirconium ion leading to slower reactant diffusion or to electronic differences in the agents causing changes in the reactivity. This finding is also in agreement with the reported literature elsewhere^84,88,89^.

### GC–MS and NMR data of degradation reactions of CWAs Sarin and Soman

**Figure 9:**
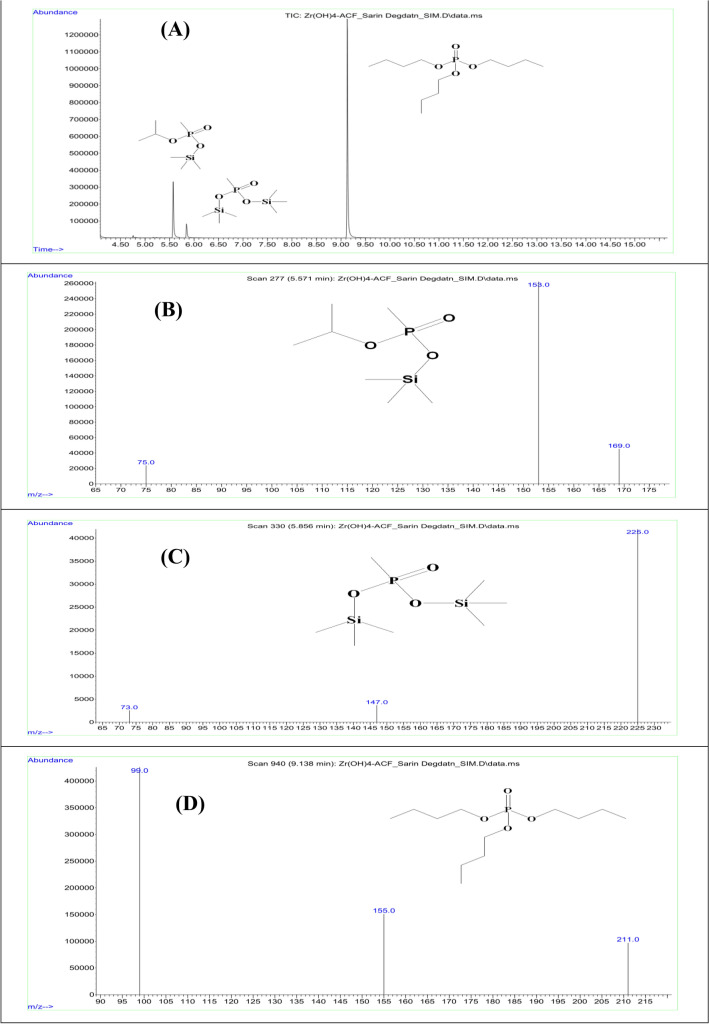
**(A)** GC–MS Chromatogram in SIM mode: BSTFA derivatization of degradation products of CWA sarin at RT 5.571 min for isopropyl trimethylsilyl methylphosphonate; RT 5.856 min for bis(trimethylsilyl) methylphosphonate; and RT 9.136 min for tributyl phosphate (a chromatographic standard), **(B)** GC–MS Spectrum in SIM mode: BSTFA derivatization of degradation products of sarin at RT 5.576 min for isopropyl trimethylsilyl methylphosphonate; **(C)** at RT 5.856 min for bis(trimethylsilyl) methylphosphonate; and **(D)** at RT 9.136 min for tributyl phosphate (a chromatographic standard).

**Figure 10:**
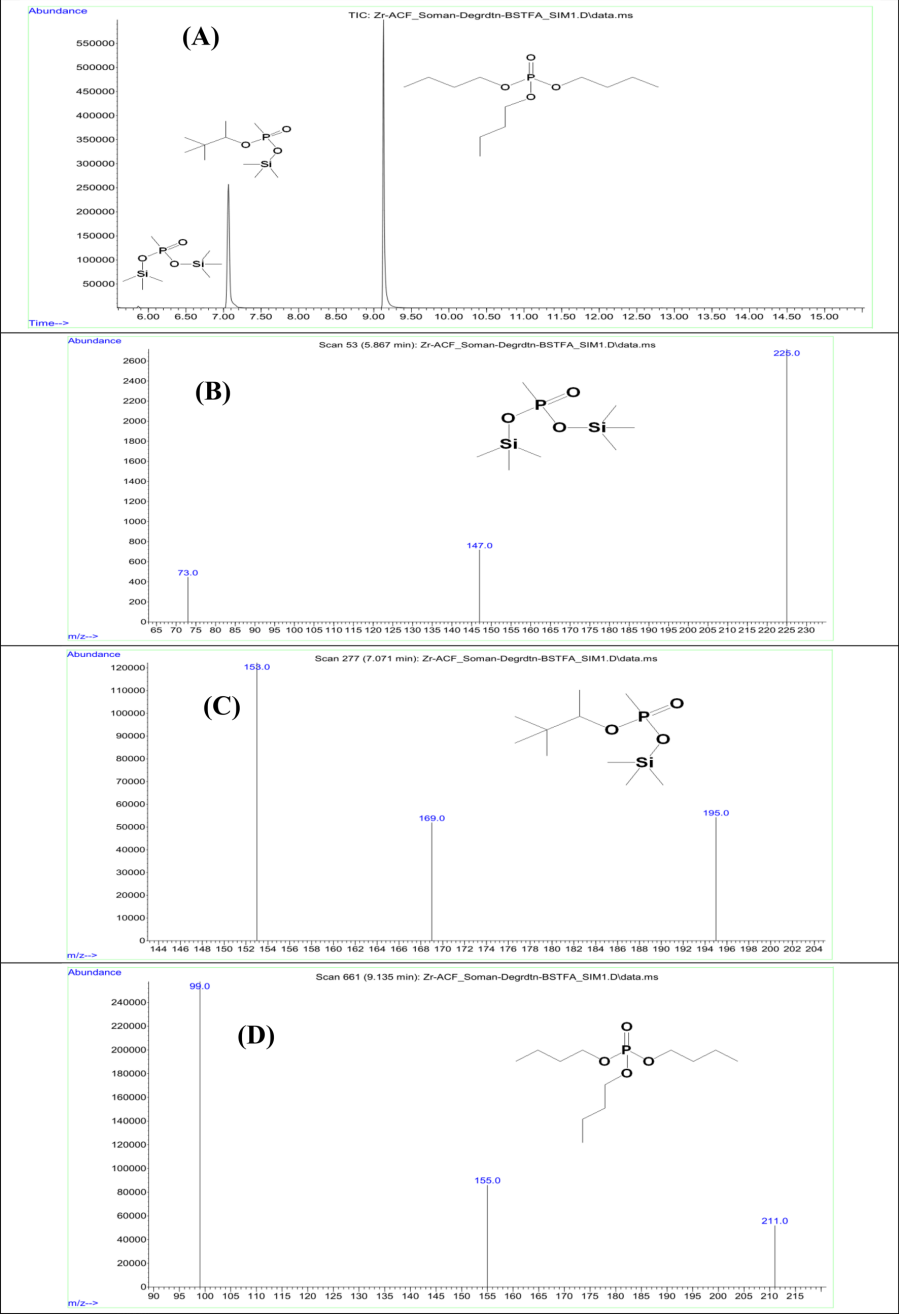
**(A)** GC–MS Chromatogram in SIM mode: BSTFA derivatization of degradation products of CWA soman at RT 5.867 min for bis(trimethylsilyl) methylphosphonate; RT 7.071 min for pinacolyltrimethylsilyl methylphosphonate; and RT 9.135 min for tributyl phosphate (a chromatographic standard), **(B)** GC–MS spectrum in SIM mode: BSTFA derivatization of degradation products of CWA soman at RT 5.867 min for bis(trimethylsilyl) methylphosphonate; **(C)** at RT 7.071 min for pinacolyltrimethylsilyl methylphosphonate; and **(D)** at RT 9.135 min for tributyl phosphate (a chromatographic standard).

In order to confirm the degradation of sarin and soman over Zr(OH)_4_@W-ACF, degradation studies were conducted. In case of sarin, the degradation products isopropyl-(trimethylsilyl) methylphosphonate (BSTFA derivative of monobasic acid) and bis(trimethylsilyl) methylphosphonate (BSTFA derivative of dibasic acid) were obtained at retention time 5.571 and 5.856 min, respectively (Fig. 9). While in soman, the degradation products pinacolyl-(trimethylsilyl) methylphosphonate (BSTFA derivative of monobasic acid) and bis(trimethylsilyl) methylphosphonate (BSTFA derivative of dibasic acid) were obtained at retention time 7.071 and 5.867 min respectively (Fig. 10).

The identification of the degradation products was also performed by using solution-state NMR spectroscopy on a Bruker AV III 600 MHz NMR spectrometer (Bruker Biospin, Fallenden, Switzerland) NMR spectrometer. The samples were irradiated and observed by using a 5 mm broadband observe (BBFO) smart probe. The data acquisition and processing were performed on Topspin 3.2 software (Bruker Biospin, Fallenden, Switzerland).

The sample obtained after elution of the residual analytes from the Zr(OH)_4_@W-ACF fabric was dissolved in the respective deuterated solvent (550 µL) and taken in a Wilmad PP-7 sample tube. The suspected products were polar or ionic, they are susceptible to a wide variation in chemical shifts the NMR experimental conditions were carefully adjusted and care was taken to maintain the similar experimental conditions (viz. sample temperature, airflow rate, composition) during all the experiments performed in a particular solvent. The probe was preheated to 25 °C (established under an airflow rate of 400 L min^−1^) and the samples were allowed to equilibrate for 5 min before each experiment. First of all screening of the samples was performed by using ^31^P{^1^H} NMR experiments (by using standard pulse program zgpg30 of Bruker pulse program library) to ascertain if any phosphorus-containing compound is present in the sample. This program performs decoupling of the proton frequencies by using Waltz16 composite pulse (pcpd2 80 µsec). The spectral width was kept at 400 ppm and the central frequency was fixed at 25 ppm to ensure uniform excitation of the spins. Each spectrum was recorded by using 32 k data points that were zero-filled to 64 k data points. To obtain good line shapes after Fourier Transformation, exponential window function and line broadening function of 0.30 Hz was also applied on the free induction decay (FID). The NMR spectra were subjected to manual phase and baseline correction.

**Figure 11:**
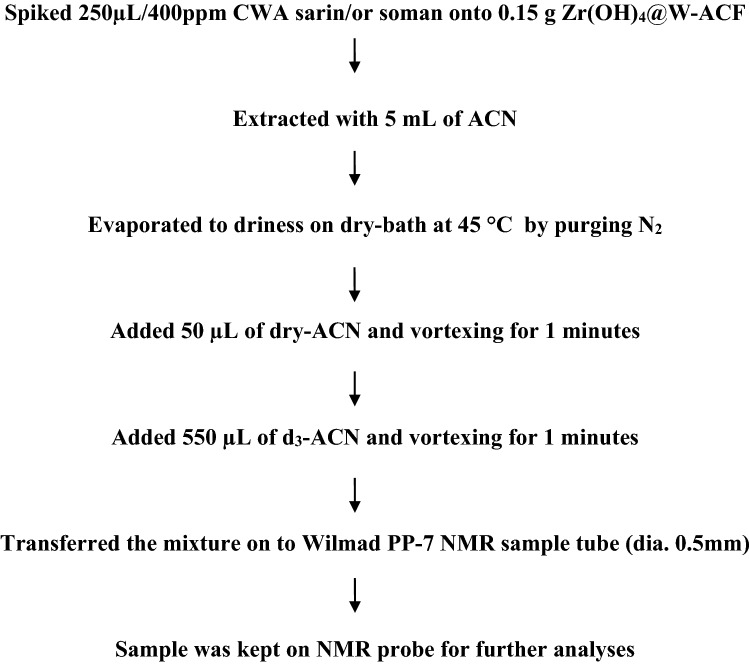
Sample preparation for NMR analyses.

**Figure 12:**
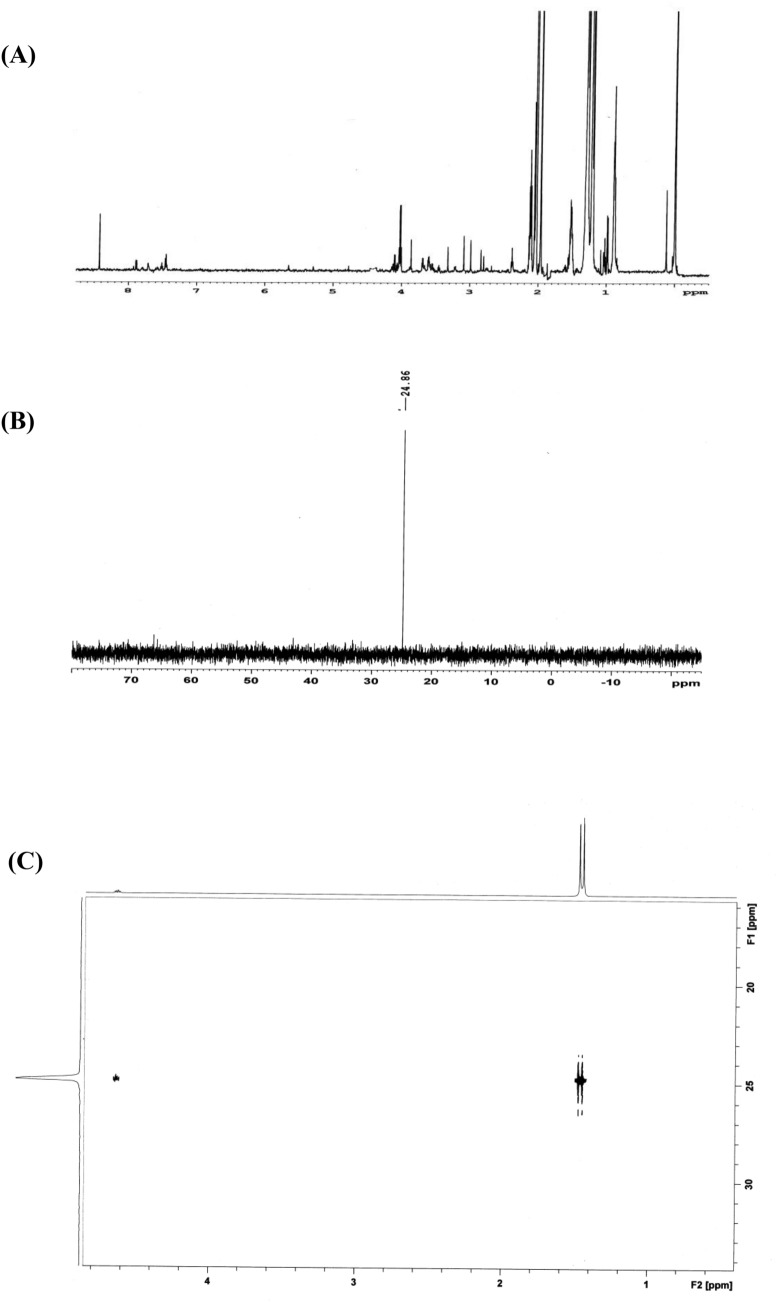
**(A)** The first (1H) NMR spectrum demonstrates the mixture of compounds obtained after the degradation of sarin. The relevant degradation product(s) cannot be identified from the spectrum. **(B)** The second (31P{1H}) NMR spectrum clearly shows the presence of one phosphorus-containing compound. Signals for sarin and the characteristic P-F coupling (1043 Hz) is missing. The chemical shift is also observed to be to be in the region corresponding to that of O-alkyl aklylphosphonic acids. **(C)** The third (31P-1H) NMR spectrum clearly shows the presence of isopropyl methylphosphonic acid (IMPA). Conclusively identification was performed by recording the spectrum standard addition of IMPA to the reaction mixture (not shown in the figure).

**Figure 13:**
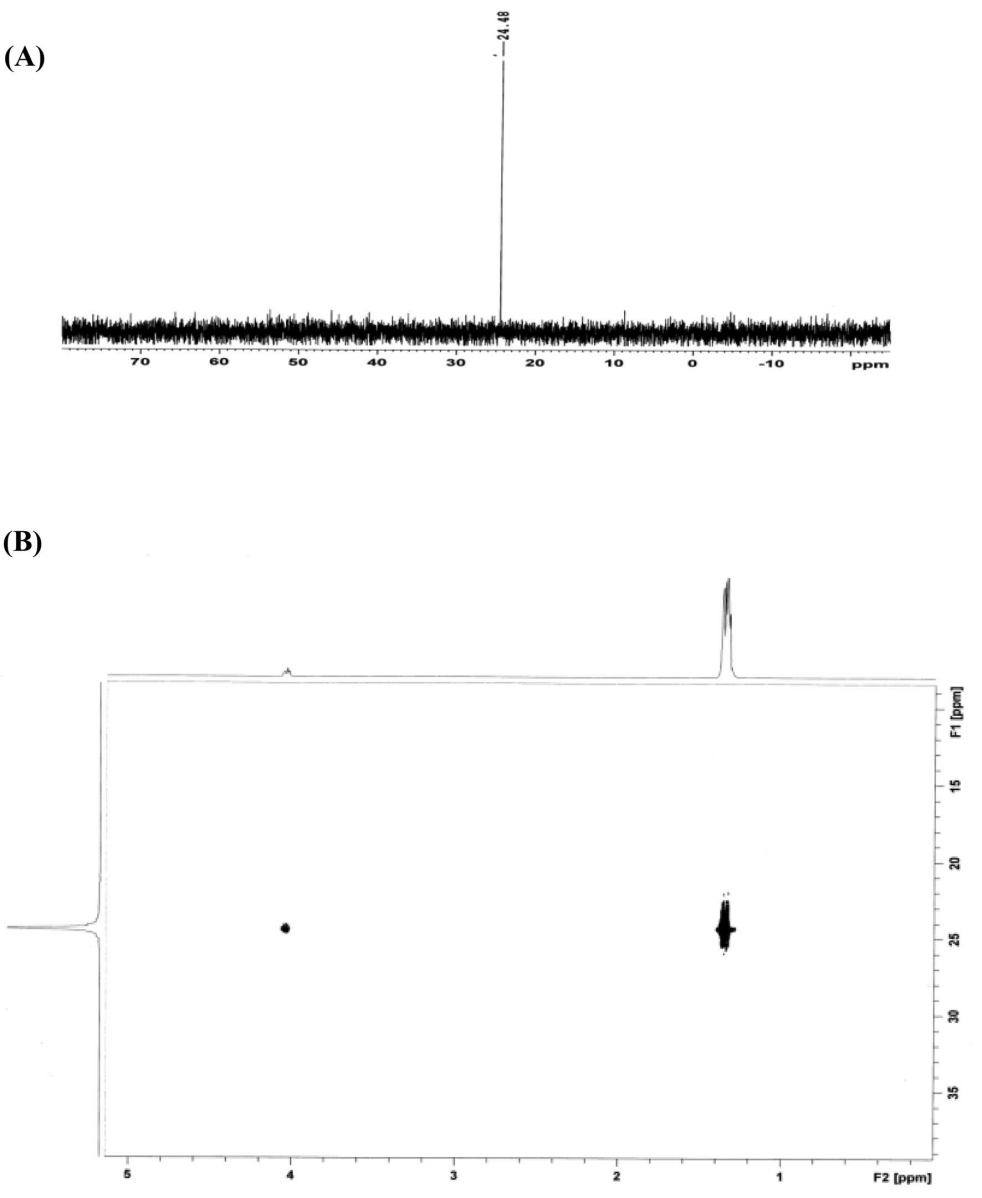
**(A)** The first (^31^P{^1^H}) NMR spectrum clearly shows the presence of only one phosphorus containing compound and the absence of soman on the reaction residue. But the chemical shift is observed to be in the region of O-alkyl alkylphosphonic acids. **(B)** The second (^31^P-^1^H) correlation NMR spectrum clearly shows the presence of pinacolyl methylphosphonic acid (PMPA). Conclusively identification was performed by recording the spectrum standard addition of PMPA to the reaction mixture (not shown in the figure).

The decontamination of sarin using Zr(OH)_4_@W-ACF was studied by NMR spectroscopy. The method adopted is presented in the flow chart Fig. 11. First of all ^1^H NMR spectrum was recorded for the sample. This experiment did not yield any information due to the intense solvent peaks. Therefore, it was followed by a ^1^H NMR with solvent suppression performed by using a shape pulsewith C-13 low power decoupling on f2 during WET solvent suppression and acquisition period (Fig. 12) from the Bruker standard pulse program library. Although a large number of proton-containing components were observed, they could not be identified or attributed to the analyte (Nerve agent sarin). Therefore, to check the decontamination efficiency, a ^31^P{^1^H} experiment was performed. The spectrum indicated a signal at 24.86 ppm i.e. the presence of phosphorus-containing analyte without the characteristic P-F coupling. The NMR parameters (chemical shifts and coupling constants) for two different nuclei possessing scalar couplings are obtained from the 2D ^1^H–^31^P HSQC/HMQC experiments. A subset of all proton signals with a scalar coupling to phosphorus of organophosphorus compound can be observed by using these experiments. Therefore, to ascertain the analyte’s identity, ^31^P-^1^H FAST-HMQC experiment^90^ was used. It filtered the irrelevant compounds and generated a correlation spectrum between the ^31^P and the scalar coupled ^1^H of the degradation product. It indicated that the products were isopropyl methylphosphonic acid (Fig. 12) and pinacolyl methylphosphonic acid (Fig. 13). Identification of a compound is achieved by comparison with a reference spectrum. The number of resonances, chemical shifts, and coupling constants was found to be consistent.

### Air permeability and tearing strength of Zr(OH)_4_@W-ACF Fabric

Taking all the above results in to consideration and to check the feasibility of this adsorbent layer for the development of CWAs protective ensemble, air permeability and tearing strength of Zr(OH)_4_@W-ACF fabric were also carried out^10^. As air permeability and tearing strength are the important characteristics of NBC permeable protective suit. Air permeability provides comfort to the wearer in warfare scenario and tear strength is required in chemical protective suit as high tear strength of textiles ensure that there should not be any leakage of CWAs if any sharp objects punctures the fabric. Air permeability helps to maintain the body temperature by allowing the air to reach and pick moisture from the surface of the body. Fabric porosity facilitates the ease of air flow across the fabric and lead to high comfort to the combatant in warfare. Fabric use in such type of application are suggested to have adequate air permeability i.e. 30 cc/cm^2^/sec^91,92^. Hence, the filter layer should have adequate air permeability so that it can release the build-up heat stress^33^. The air permeability of Zr(OH)_4_@W-ACF and control fabric was measured according to ASTM D73^93^. As can be seen from the Table 3, the air permeability of Zr(OH)_4_@W-ACF and control sample are having 60 cc/cm^2^/sec and 15,000 cc/cm^2^/sec, respectively which shows after impregnation of W-ACF cloth even though air permeability reduced however it met the specified limit of the requirement of chemical protective suit.

**Table 3:** Air-permeability and tearing-strength of W-ACF Control and Zr(OH)_4_@W-ACF.

Sample name	Air-permeability@12.7 mm WG (cc/cm^2^/sec)	Tearing-strength (gf)
W-ACF	15,000	0.0096
Zr(OH)_4_@W-ACF	60	0.0192

Tear strength of Zr(OH)_4_@W-ACF and control fabric was also measured. Materials having low tear strength can tend to have poor resistance to the abrasion and even a small abrasion lead to tear the sample and make the easy entrance of CWAs through the fabric which defeats the total purpose of NBC protection. It has been found that after the impregnation of Zr(OH)_4_ over W-ACF the tear strength of fabric is increased from 0.0096 gf to 0.0192 gf, hence the impregnation leads to improve the fabric in terms of tear strength and this kind of fabric is utmost required while in the development of protective textile which is life saving device.

### Nerve agent permeation test of of Zr(OH)_4_@W-ACF fabric

**Figure 14:**
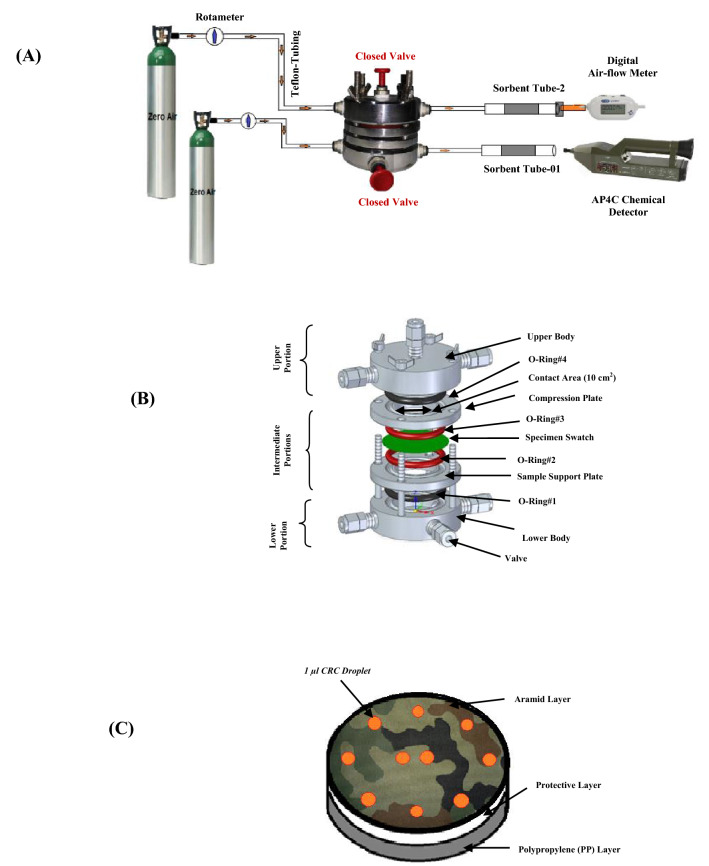
The schematic representations- **(A)** CWAs Permeation Test setup/or Facility; **(B)** Devised Permeation Test-Apparatus; and **(C)** Pattern for distribution of droplets of chemicals related to chemical weapons convention (CRC’s) over the composite-swatch of W-ACF/or modified fabric (Actual images and draw using MS paint).

In order to explore the suitability of the prepared composite fabric of Zr(OH)_4_@W-ACF as a core filter layer for the development of NBC protective ensemble, nerve agent permeation study was carried out. This test was in accordance with US Military standard. The test method (TOP 08–2-501A: 2013, Washington, D.C.)^79^ describe the test operating procedure for permeation testing of swatch of test materials with CWAs or their simulants. This test procedure is suggested to verifying the protective feasibility of the ensemble devices, when it directly exposed with CWAs on its surface in liquid or vapor phase. In this context, a permeation test study was performed with our developed test facility as depicted in Fig. 14 Bureau of Indian Standards (IS 17380: 2020). To perform this test, a three layer swatch having diameter 3.5 cm consists of (a) top most aramid layer, having camouflage patter; (b) middle one is protective layer (i.e. Zr(OH)_4_@W-ACF or control W-ACF); and (c) bottom layer (i.e. polyproplylene non-woven) was drawn. The CWA sarin was uniformly distributed on swatch sample using calibrated microsyringe in such a way that sarin droplets of size approx 1 μl. The chambers were closed and adsorbent tubes filled with XAD-2 were placed in lower cups and empty adsorbent tubes at upper cups. The amount of penetrated agent was collected in adsorbent filled tubes. At the end of the test, the amount of agent in the adsorbent filled tubes was determined by extracting the adsorbent material. The extracted samples were analysed by GC–MS with respect to standard calibration plot and it was summarized in Table 4.

**Table 4:** Nerve agent permeation of W-ACF Control and Zr(OH)_4_@W-ACF.

Sample name	Permeation amount (µg/cm^2^)	Specified value (µg/cm^2^)
Zr(OH)_4_@W-ACF #Sarin	0.027705	≤ 4.0
W-ACF_Control #Sarin	13.81	≤ 4.0

As can be seen from the Table 4 in case of Zr(OH)_4_@W-ACF (5 samples of each) the nerve agent penetration was found only 0.027 ± 0.002 µg/cm^2^ while control sample has 13.81 ± 1.2 µg/cm^2^ nerve agent penetration. The specified limit for NBC suit is 4 µg/cm^2^. Hence, from the above results it can be concluded that the resulting developed composite fabric which amalgamate the characteristics of reactive Zr(OH)_4_ and high surface area of W-ACF i.e. Zr(OH)_4_@W-ACF will be a good candidate as a core layer for the development of NBC protective ensemble.

## Conclusions

Herein, we have demonstrated the self detoxifying capability of Zr(OH)_4_@W-ACF as an adsorbent layer for the degradation of CWAs sarin and soman and feasibility study for the development of next generation protective ensemble utlizing Zr(OH)_4_@W-ACF fabric as core filter layer was also explored. The Zr(OH)_4_@W-ACF material relies on the high surface area of ACF which directs the unique growth of Zr(OH)_4_ particles and enables effective capturing of CWAs followed by destruction into innocuous substances which exemplify the use of textile and material together as new generation textile for development of protective gears. Results of several characterization techniques such as FT-IR, EDX, XPS, Raman and SEM results confirmed the in-situ deposition of Zr(OH)_4_ on the surface of W-ACF_._ The CWA sarin degradation efficiency of the Zr(OH)_4_@W-ACF leads to ninefold enhancement in comparison to its static counterpart. The kinetics of the in-situ degradation of CWAs sarin and soman on the surface of Zr(OH)_4_@W-ACF follows the first-order kinetics. The half-lives for sarin and soman were calculated and found to be 2.84 min and 30 min, respectively. The rate constant was found to be 0.244 min^−1^ and 2.31 × 10^–2^ min^−1^ for sarin and soman, respectively. The degradation products of sarin and soman were analyzed and confirmed with NMR, and GC–MS analysis. The suitability of prepared reactive composite fabric of Zr(OH)_4_@W-ACF as a core filter layer for the development of protective suit was also tested, and it was found to be meeting the specified criteria in terms of air permeability, tearing strength and nerve agent permeation as per TOP 08–2-501A: 2013 and BIS standard IS 17380:2020. The coupling of high adsorption capability of ACF and reactive behaviour of Zr(OH)_4_ offers an attractive platform for combating CWA defense in particular important for the development of CWA protective suits with low thermal burden, enhanced flexibility, and self detoxifying capability which open up the new horizon in the field of new generation CWA protective clothing and thus find diverse application in defence and environmental applications.

## Supplementary Information

Supplementary Information.
