# Supplementary material for: In-situ detoxification of schedule-I chemical warfare agents utilizing Zr(OH)4@W-ACF functional material for the development of next generation NBC protective gears
Source: Sci Rep. 2021 Dec 24;11:24421. doi: 10.1038/s41598-021-03786-8 (PMC8709862; doi:10.1038/s41598-021-03786-8)
Supplement: Supplementary file 1 — Supplementary Information. [file 41598_2021_3786_MOESM1_ESM.docx]

***In-situ* Detoxification of Schedule-I Chemical Warfare Agents Utilizing Zr(OH)_4_@W-ACF Functional Material for the Development of Next Generation NBC Protective Gears**

Mohammad Imran, Virendra V Singh^*^, Prabhat Garg, Avik Mazumder, Lokesh K. Pandey, Pushpendra K. Sharma, Jyotiranjan Acharya, Kumaran Ganesan

Defence Research & Development Establishment, DRDO, Jhansi Road, Gwalior-474002, India

^*^E-mail: [vvs17@drde.drdo.in](mailto:vvs17@drde.drdo.in)

**Fig S1: TGA curve of ZrOCl_2_.8H_2_O.**

**Fig S2: XRD pattern of W-ACF.**

.

**Fig S3: XPS survey spectra of (A) ACF (B) Zr(OH)_4_@W-ACF, deconvoluted peaks of Zr(OH)_4_@W-ACF (C) Zr 3d (D) C 1s and (E) O 1s region.**

**Fig S4: Raman Spectra of (a) W-ACF and (B) Zr(OH)_4_@W-ACF.**


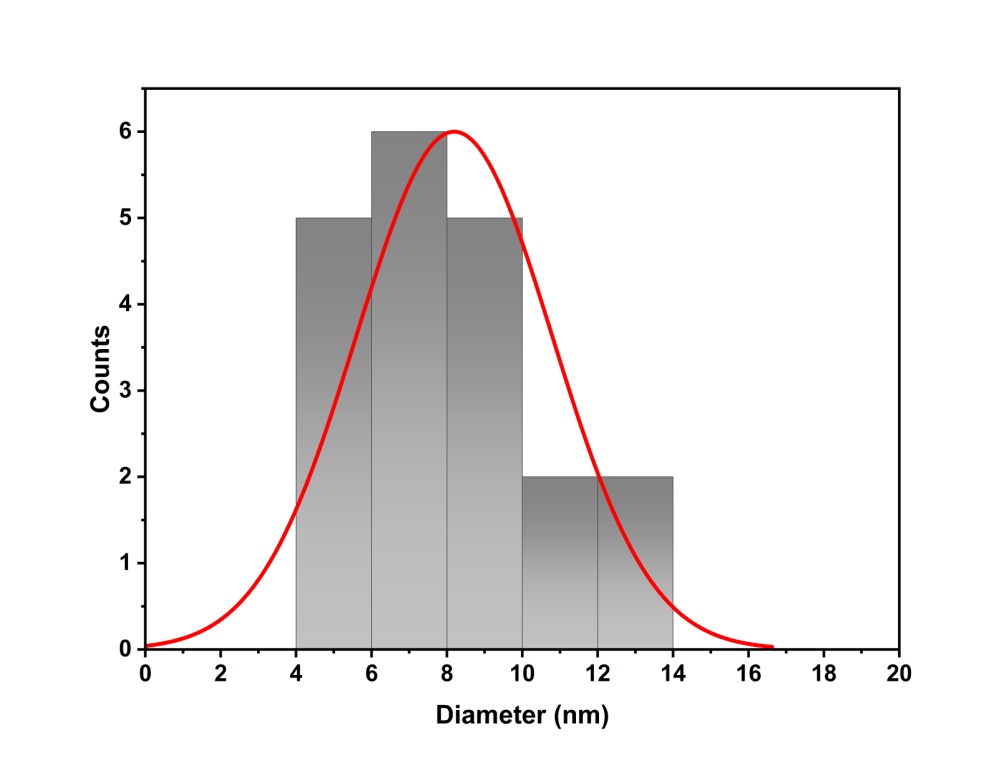


**Fig S5: Histogram of particle size distribution from TEM measurement**

**Fig S6: Optimization of organic solvent on extraction efficiency of sarin.**

**Fig S7: Optimization of extraction time with ethyl acetate on extraction efficiency of sarin.**

**Fig S8: Degradation of sarin on NaOH@W-ACF at ambient temperature.**
